# Microfluidic-Based Technique for Measuring RBC Aggregation and Blood Viscosity in a Continuous and Simultaneous Fashion

**DOI:** 10.3390/mi9090467

**Published:** 2018-09-14

**Authors:** Yang Jun Kang

**Affiliations:** Department of Mechanical Engineering, Chosun University, 309 Pilmun-daero, Dong-gu, Gwangju 61452, Korea; yjkang2011@chosun.ac.kr; Tel.: +82-62-230-7052

**Keywords:** red blood cell (RBC) aggregation, blood viscosity, continuous and simultaneous fashion, microfluidic device, RBC aggregation index, in vitro fluidic circuit

## Abstract

Hemorheological properties such as viscosity, deformability, and aggregation have been employed to monitor or screen patients with cardiovascular diseases. To effectively evaluate blood circulating within an in vitro closed circuit, it is important to quantify its hemorheological properties consistently and accurately. A simple method for measuring red blood cell (RBC) aggregation and blood viscosity is proposed for analyzing blood flow in a microfluidic device, especially in a continuous and simultaneous fashion. To measure RBC aggregation, blood flows through three channels: the left wide channel, the narrow channel and the right wide channel sequentially. After quantifying the image intensity of RBCs aggregated in the left channel (<I_RA_>) and the RBCs disaggregated in the right channel (<I_RD_>), the RBC aggregation index (AI_PM_) is obtained by dividing <I_RA_> by <I_RD_>. Simultaneously, based on a modified parallel flow method, blood viscosity is obtained by detecting the interface between two fluids in the right wide channel. RBC aggregation and blood viscosity were first evaluated under constant and pulsatile blood flows. AI_PM_ varies significantly with respect to blood flow rate (for both its amplitude and period) and the concentration of the dextran solution used. According to our quantitative comparison between the proposed aggregation index (AI_PM_) and the conventional aggregation index (AI_CM_), it is found that AI_PM_ provides consistent results. Finally, the suggested method is employed to obtain the RBC aggregation and blood viscosity of blood circulating within an in vitro fluidic circuit. The experimental results lead to the conclusion that the proposed method can be successfully used to measure RBC aggregation and blood viscosity, especially in a continuous and simultaneous fashion.

## 1. Introduction

Cardiovascular diseases (CVDs), including stroke, coronary heart disease, and myocardial infarction, have been regarded as the most common causes of global deaths [1]. Because vascular blockage or blood clotting occurs without symptoms or warning, it usually causes serious complications or sudden deaths. However, the biochemical properties of blood, including biomarkers [2], cholesterol, and glucose, do not provide sufficient clues for the early detection of CVDs [3]. Because a strong relationship between CVDs and hemorheological properties was reported [4,5,6], the biophysical properties of blood have been employed to monitor or screen patients with CVDs. Hemorheological properties are affected by several factors, including the hematocrit (Hct), cell components (red blood cells, white blood cells, and platelets), the cell-free layer, and plasma proteins [7,8]. To measure the rheological properties of blood circulating under in vitro [9] or ex vivo [10,11] conditions, large volumes of blood are collected from closed circuits at intervals with a specific duration. Then, bulk-sized instruments are employed to measure hemorheological properties under in vitro conditions. The different sizes between in vivo blood vessels (e.g., in the hind limb of a dog) and in vitro instruments (e.g., an Oswald viscometer) cause differences in blood viscosity [12]. Because blood collection causes a decrease in blood volume in closed loops, a lactated Ringer’s solution can be added to maintain a sufficient blood volume [13,14]. This hemodilution procedure continuously alters the hematocrit levels and fibrinogen concentrations in plasma. In addition, because red blood cells (RBCs) become adhered to the non-physiological surface of the tubes and are exposed to high shear stress, RBC hemolysis increases over time [14]. Thus, it is significant to quantify these variations of the biophysical properties of blood, especially in a continuous and simultaneous fashion. Microfluidic devices have several merits, such as small volume consumption, fast response, easy handling and disposability for point-of-care testing. For this reason, they have been widely applied to quantify hemorheological properties.

First, RBC aggregation is affected by several factors, including membrane deformability, hematocrit levels, plasma proteins, and shear rate [15]. To evaluate the RBC aggregation of blood, a microfluidic channel is filled with blood. Then, to induce the aggregation or disaggregation of RBCs, the blood flow is agitated or stopped by using several devices, including pinch valves [15], syringe pumps [16,17], air-suction pumps [18], magnetic bars [19], and vacuum pumps [20]. As summarized in Table 1, several methods, such as photometric intensity measurements (light transmission or light backscattering) [15,19,20], electric impedance measurements [21,22], ultrasonic speckle imaging [23], and microscopic imaging [9,16,17,18,24,25,26,27], have been suggested to obtain a typical syllectogram (a signal vs. time plot). Subsequently, the conventional RBC aggregation index (AI_CM_) is obtained by analyzing the syllectogram. Because the aforementioned methods were demonstrated by stopping the blood flow, the RBC aggregation index was quantified at intervals of 240–400 s [9,16,17,18,24]. For this reason, these methods do not apply for quantifying the RBC aggregation index in a continuous blood flow.

Secondly, because RBC aggregation is significantly affected by several factors, it is necessary to determine why RBC aggregation varies. For this reason, blood viscosity measurements can be employed to evaluate hemorheological properties, including the hematocrit, RBC deformability, RBC aggregation, and plasma proteins. At higher shear rates, blood viscosity varies depending on the degree of RBC deformability. However, at low shear rates, blood viscosity is determined by RBC aggregation. The hematocrit and plasma proteins contribute to significantly increase blood viscosity. To measure blood viscosity in a microfluidic device, blood is made to flow at a specific flow rate or velocity using several driving sources, such as syringe pumps, pressure sources [28], surface tension [29,30,31,32], and pipettes [33]. After that, as shown in Table 1, several measurement techniques, including co-flowing streams [34], modified parallel flows [9,24], microflow compartments [35,36], reversal flow-switching [10,37,38,39], advancing meniscus [28,29,30,31,32,33], and electric impedance measurements [40,41], can be used to measure blood viscosity. Considering some critical issues, such as RBC clogging and precise flow-rate control and calibration, the modified parallel flows method (MPFM) [9,24] shows high potential for continuous measurement of blood viscosity. Previously, our group demonstrated that RBC aggregation and blood viscosity can be simultaneously measured at specific intervals [9,24]. However, because the blood flow has to be agitated or stopped in order to measure RBC aggregation, the blood flow rate was controlled by periodically turning a syringe pump on and off. For this reason, it is fundamentally necessary to develop a new measurement method for measuring RBC aggregation, particularly for continuous blood flows. There is a need for a new method that has the ability to measure RBC aggregation and blood viscosity in a simultaneous and continuous fashion.

In this study, a method for the continuous and simultaneous measurement of RBC aggregation and blood viscosity is proposed, made possible by analyzing blood flow in a microfluidic device.

To demonstrate this method, a microfluidic device, composed of a left wide channel (width = 2000 µm), a narrow channel (width = 100 µm), a right wide channel (width = 2000 µm), inlets, and outlets, was used. To measure RBC aggregation in a continuous blood flow, blood sequentially flowed through the three channels with different widths (the left wide channel, the narrow channel, and the right wide channel). When blood flows in the left wide channel, RBC aggregation occurs because of the lower shear rates.

Next, when blood flows into the narrow channel, the RBC aggregates are completely broken owing to the high shear rates. Finally, when blood flows into the right wide channel, RBCs tends to aggregate over time because of lower shear rates. Simultaneously, by adding a phosphate buffered saline (PBS) solution as a reference fluid into the right wide channel using a syringe pump, blood viscosity can be continuously measured by detecting interfacial locations between blood and the PBS solution over time. 

The proposed method has several advantages when compared with previous methods. First, RBC aggregation is measured continuously over time. Specifically, when compared with a previous method [9], the proposed method does not require an on-off control of the blood flow using pinch valves, so that RBC aggregation can be quantified continuously rather than at specific time intervals. Secondly, blood viscosity can also be evaluated continuously over time. During RBC aggregation measurements, blood viscosity can be used to determine the potential reasons for the measured changes in RBC aggregation. First, as a demonstration of the performance of the proposed method, variations in the RBC aggregation index and blood viscosity were simultaneously obtained by varying a constant blood flow rate and using various concentrations of a dextran solution. Then, the RBC aggregation index obtained via the proposed method was quantitatively compared with those obtained via previously mentioned methods. Secondly, under a pulsatile blood flow, variations in the measured RBC aggregation index and blood viscosity were obtained by modifying several factors, including period (T), hematocrit (Hct), and the base solution as the dextran solution. Finally, for blood circulating within an in vitro fluidic circuit, the variations in the RBC aggregation index and blood viscosity were evaluated in a continuous and simultaneous fashion.

## 2. Materials and Methods

### 2.1. Blood Sample Preparation

In accordance with the ethics committee of the Chosun University Hospital (CUH), all experiments were performed ensuring that the procedures were appropriate and humane. Concentrated RBCs were purchased from the Gwangju–Chonnam blood bank (Gwangju, Korea) and stored at 4 °C. When the storage time of RBCs exceeded seven days, all RBCs were removed. After a washing procedure was performed twice, pure RBCs were collected from the concentrated RBCs. Blood samples were prepared by adding RBCs into specific dextran solutions. Thereafter, the blood samples were kept at 4 °C before the blood tests. All experiments were completed within four hours.

First, to evaluate the effects of the hematocrit on RBC aggregation and blood viscosity, the hematocrit of normal blood (Hct = 30% and 50%) was prepared by adding normal RBCs into a PBS solution (1×, pH 7.4, GIBCO, Life Technologies, Seoul, Korea) or into dextran solutions with specific concentrations. To stimulate RBC aggregation, the specific concentration of dextran solution was selected by referring to previous works [9,16,18,24]. More specifically, four different concentrations of the dextran solution (C_dextran_ = 5 mg/mL, 10 mg/mL, 15 mg/mL, and 20 mg/mL) were prepared by mixing dextran (*Leuconostoc* spp., MW = 450–650 kDa, Sigma-Aldrich, St. Louis, MO, USA) with the PBS solution. According to previous works, RBC aggregation increased gradually by increasing the concentration of dextran solution.

### 2.2. Fabrication of a Microfluidic Device and Experimental Procedure

**Figure 1:**
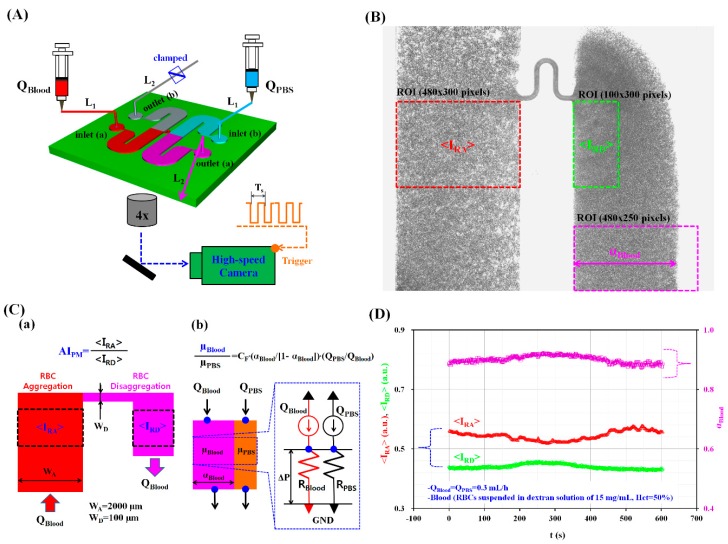
A proposed method for measuring red blood cells (RBCs) aggregation and blood viscosity in a continuous and simultaneous fashion. (**A**) Schematic diagram of an experimental setup including a microfluidic device, two syringe pumps, and microscopic image acquisition system with a high-speed camera. (**B**) Specific regions of interest (ROIs) for calculating three parameters: the image intensity of RBCs aggregated in the left wide channel (<I_RA_>), the RBCs disaggregated in the right wide channel (<I_RD_>) and the blood-filled width in the right wide channel (α_Blood_) from microscopic images. (**C**) A proposed method for measuring RBC aggregation index (AI_PM_) and blood viscosity (µ_Blood_). (a) The RBC aggregation index (AI_PM_) was evaluated by dividing <I_RA_> by <I_RD_>) (AI_PM_ = <I_RA_>/<I_RD_>). (b) The µ_Blood_ was quantified by monitoring α_Blood_ in the right wide side channel, at a specific flow-rate ratio of phosphate buffered saline (PBS) solution to blood (Q_PBS_/Q_Blood_). GND denotes zero value of gauge pressure (i.e., P = 0). (**D**) As a preliminary demonstration, blood (Hct = 50%, RBCs suspended in dextran solution (C_dextran_ = 15 mg/mL)) and PBS solution were supplied into the microfluidic device at the same flow rate of 0.3 mL/h. The three parameters <I_RA_>, <I_RD_> and α_Blood_ were obtained for a specific duration of 600 s.

Figure 1A shows a schematic diagram of our experimental setup, which included a microfluidic device, two syringe pumps, and a microscopic image acquisition system combined with a high-speed camera.

The microfluidic device used for measuring RBC aggregation and blood viscosity was composed of two wide channels (width = 2000 μm and length = 22.5 mm), two inlets (a, b), and two outlets (a, b). The left and right wide channels consisted of an inlet, a straight channel, and an outlet. The middle position of two wide channels was connected with a narrow channel (width = 100 μm and length = 1750 μm). The channel depth of the microfluidic device was set to 100 μm. 

A silicon master mold was fabricated using conventional micro-electromechanical system fabrication techniques, including photolithography and deep reactive-ion etching. Polydimethylsiloxane (PDMS) (Sylgard 184, Dow Corning, Midland, MI, USA) was mixed with a curing agent at a ratio of 10:1. This PDMS mixture was poured onto the silicon master mold, which was placed in a Petri dish. The air bubbles dissolved in PDMS were completely removed by operating a vacuum pump for 1 h. After curing the PDMS in a convective oven at 70 °C for 1 h, the PDMS block was peeled off from the silicon master mold. The PDMS block was cut with a razor blade. Four ports were punched with a biopsy punch (outer diameter = 0.75 mm). After treating the PDMS block and a glass substrate with oxygen plasma (CUTE-MPR, Femto Science Co., Gyeonggi, Korea), the microfluidic device was completed by bonding the PDMS block to the glass substrate.

Two polyethylene tubes, L_1_ (length = 300 mm and inner diameter = 250 μm) and L_2_ (length = 200 mm and inner diameter = 250 μm), were tightly fitted to the inlet ports and outlet ports, respectively. To remove air bubbles in the microfluidic channels and avoid the non-specific binding of plasma proteins to the inner surface of the microfluidic channels, a bovine serum albumin (BSA) solution with a concentration of C_BSA_ = 2 mg/mL was delivered through outlet (a) of the microfluidic device using a disposable syringe. After an elapse of 5 min, all microfluidic channels were filled with the PBS solution using a disposable syringe. Subsequently, outlet (b) was completely clamped with a pinch valve.

After two disposable syringes (~1 mL) were filled with blood and PBS, they were both connected to the ends of tube L_1_. Subsequently, each syringe was installed into the corresponding syringe pump (neMESYS, Centoni GmbH, Korbussen, Germany). By setting the two syringe pumps at the same flow rate (Q_Blood_ = Q_PBS_), the blood and the PBS solution were simultaneously delivered to inlet (a) and inlet (b), respectively.

The microfluidic device was positioned on an optical microscope (BX51, Olympus, Tokyo, Japan) equipped with a 4× objective lens (Numerical Aperture (N.A.) = 0.1). A high-speed camera (FASTCAM MINI, Photron, San Jose, CA, USA) was used to capture microscopic images of the blood flow in the microfluidic channels. The camera had a spatial resolution of 1280 × 1024 pixels. Each pixel corresponded to 10 µm. Using a function generator (WF1944B, NF Corporation, Tokyo, Japan), a periodic pulsed signal (T_s_ = 50 ms or 1 s) triggered the high-speed camera. Microscopic images were then sequentially captured at a frame rate of 1 kHz. All experiments were conducted at a room temperature of 25 °C.

### 2.3. Quantification of Image Intensity and Blood Flow-Rate

To measure RBC aggregation and blood viscosity, the variations of three parameters (<I_RA_>, <I_RD_>, and α_Blood_) were obtained by analyzing microscopic images of the blood flow in the left and right wide channels. Here, α_Blood_ denotes blood-filled width in the right wide channel. First, as shown in Figure 1B, to evaluate the RBC aggregation in a continuous blood flow, a region of interest (ROI, 484 × 300 pixels) was selected within the left wide channel. By conducting digital image processing with a commercial software package (MATLAB R2014a, MathWorks, Natick, MA, USA), the averaged image intensity of RBC aggregation (<I_RA_>) was obtained by averaging the image intensity values of RBCs distributed within the specified ROI of the left wide channel. Additionally, to quantify RBC disaggregation in continuous blood flows, a specific ROI (100 × 300 pixels) was selected within the right wide channel. The averaged image intensity of RBC disaggregation (<I_RD_>) was obtained by averaging the image intensity values of RBCs distributed within the specified ROI of the right left wide channel.

Secondly, blood viscosity was obtained by quantifying the interfacial locations between the blood and the PBS solution in the right wide channel. To obtain the blood-filled width (α_Blood_) in the right wide channel, the obtained gray images were converted into binary images using Otsu’s method [42]. By calculating the arithmetic average over the ROI (480 × 250 pixels), the blood-filled width (α_Blood_) over time was obtained.

Finally, to quantify the blood flow rate supplied from the fluidic divide (FD) into the microfluidic channel, the velocity fields of the blood flow in the left wide channel were obtained by employing a time-resolved micro-PIV (particle image velocimetry) technique. The size of the interrogation window was 64 × 64 pixels and the window overlap was 50%. The obtained velocity fields were validated using a median filter. The averaged blood velocity (<U>) was then obtained by averaging the blood velocity fields over a specific ROI (480 × 300 pixels) selected in the left wide channel. Thereafter, the blood flow-rate (Q_µPIV_) was evaluated by multiplying the averaged velocity (<U>) by the cross-sectional area of the rectangular channel (A_c_) (A_c_ = width × depth, Q_µPIV_ = <U> × A_c_).

### 2.4. Simultaneous Measurement of RBC Aggregation and Blood Viscosity in Continuous Blood Flows

In this study, the RBC aggregation index (AI_PM_) and blood viscosity (µ_Blood_) were simultaneously obtained in continuous blood flows. As shown in Figure 1C-a, under a specific blood flow-rate (Q_Blood_), RBC aggregation occurred in the left wide channel (W_A_ = 2000 µm) owing to the lower shear rates. However, in the narrow channel (W_D_ = 100 µm), the shear rate increased significantly (over 20 times) when compared with the shear rate estimated in the left wide channel. Thus, when blood passed through the narrow channel, the RBC aggregates were fully broken. As shown in Figure 1B, from the outlet of the narrow channel, RBC disaggregation was still observed within a specific region of the right channel. When compared with the image intensity of RBC aggregates in the left channel (<I_RA_>), the image intensity of the RBCs disaggregated within the specific ROI of the right channel (<I_RD_>) decreased owing to RBC disaggregation. However, at the lowest position of the right wide channel, RBCs aggregated again, as evidenced by the fact that image intensity increased. For this reason, the specific ROI for RBC disaggregation was carefully selected from the outlet of the narrow channel. Specifically, the size of the ROI for RBC disaggregation was much smaller than that for RBC aggregation. Without stopping the continuous flow of blood, RBC aggregation and disaggregation were monitored via the temporal variations of <I_RA_> and <I_RD_> simultaneously. Then, the RBC aggregation index (AI_PM_) was quantified as a new index by dividing <I_RA_> by <I_RD_> (AI_PM_ = <I_RA_>/<I_RD_>).

As shown in Figure 1C-b, the right wide channel was partially filled with blood and PBS solution. The right wide channel consisted of two streams, namely a blood stream and a PBS stream. The flow rates of blood and PBS solution are denoted as Q_Blood_ and Q_PBS_, respectively. The temporal variations of the blood-filled width (α_Blood_) were obtained to evaluate blood viscosity (µ_Blood_) over time. To simplify the mathematical model used, the right wide channel was divided into two independent channels (a blood channel and a PBS channel). More specifically, the right wide channel was simply modeled with fluidic circuit elements, such as fluidic resistances (R_Blood_, R_PBS_), and flow rates (Q_PBS_, Q_Blood_). Here, R_Blood_ and R_PBS_ denote the fluidic resistances of the blood and PBS channels, respectively. Using the same pressure drop for each channel (ΔP = R_PBS_ × Q_PBS_ = R_Blood_ × Q_Blood_), the fluidic resistance ratio of the blood channel to the PBS channel (R_Blood_/R_PBS_) is expressed as R_Blood_/R_PBS_ = Q_PBS_/Q_Blood_. For a rectangular channel with a lower aspect ratio (AR = depth/width = 100/2000), the fluidic resistance for each channel can be approximately expressed as R = 12 × µ × l/(w × h^3^), as explained in previous work [43]. Because each fluid channel has identical dimensions of channel depth (h) and length (l), an analytical formula for blood viscosity (µ_Blood_) can be simply derived as [43]:

(1) $$\;\frac{\mu_{\mathrm{Blood}}}{\mu_{\mathrm{PBS}}}=\left(\frac{\alpha_{\mathrm{Blood}}}{1-\alpha_{\mathrm{Blood}}}\right)\times \left(\frac{Q_{\mathrm{PBS}}}{Q_{\mathrm{Blood}}}\right)\;$$

However, when compared with a real fluidic channel, the mathematical model includes different boundary conditions. To compensate for these differences in the boundary conditions between real fluidic channels and our mathematical model, a specific correction factor should be included in Equation (1). Then, the formula for blood viscosity can be simply derived as:

(2) $$\;\frac{\mu_{\mathrm{Blood}}}{\mu_{\mathrm{PBS}}}\;={\;C}_{F}\times \left(\frac{\alpha_{\mathrm{Blood}}}{1-\alpha_{\mathrm{Blood}}}\right)\times \left(\frac{Q_{\mathrm{PBS}}}{Q_{\mathrm{Blood}}}\right)\;$$

According to previous studies [9,24], this approach was named the modified parallel flow method (MPFM). For a rectangular channel (width = 2000 µm and depth = 100 µm) [9], the correction factor (C_F_) was reported as C_F_ = 34.041α_Blood_^4^ − 91.75α_Blood_^3^ + 92.393α_Blood_^2^ − 40.977α_Blood_ + 7.7319. The MPFM has the ability to measure liquid and blood consistently when compared with previous methods such as a cone-and-plate viscometer or the flow-switching method [37].

As shown in Figure 1D and as a preliminary demonstration, a blood sample (Hct = 50%) was prepared by adding normal RBCs into a dextran solution with a specific concentration (C_dextran_ = 15 mg/mL). The blood and the PBS solution were simultaneously supplied into the microfluidic device at the same flow rate of 0.3 mL/h (Q_Blood_ = Q_PBS_ = 0.3 mL/h). By conducting digital image processing, the three aforementioned parameters, <I_RA_>, <I_RD_> and α_Blood_, were obtained over 600 s. Owing to RBC aggregation in the left wide channel, <I_RA_> showed higher values than <I_RD_>. Additionally, the blood-filled width (α_Blood_) varied depending on RBC aggregation. From this preliminary demonstration, the proposed method showed promising potential for simultaneously measuring RBC aggregation and blood viscosity, especially in continuous blood flow.

## 3. Results and Discussion

### 3.1. Determination of Channel Widths for Inducing and Disaggregating RBCs under Constant Value of Blood Flow Rates

**Figure 2:**
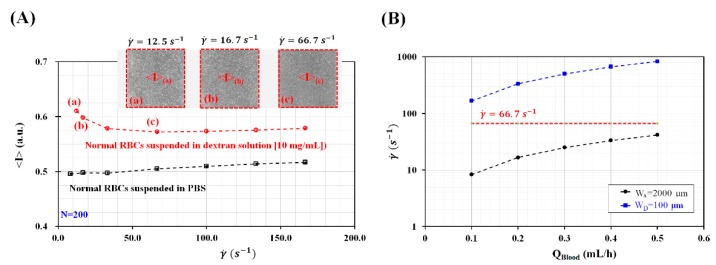
Determination of the widths of the wide and narrow channels. (**A**) Variations of image intensity (<I>) with respect to shear rate ($\dot{\gamma }$). Inset showed microscopic images captured with respect to shear rate ((a) $\dot{\gamma }=12.5{\;s}^{-1}$, (b) $\dot{\gamma }=16.7{\;s}^{-1}$, and (c) $\dot{\gamma }=66.7{\;s}^{-1}$ ). (**B**) Variations of shear rate ($\dot{\gamma }$ ) of wide channel (W_A_ = 2000 μm) and narrow channel (W_D_ = 100 μm) with respect to blood flow rate (Q_Blood_) (Q_Blood_ = 0.1, 0.2, 0.3, 0.4, and 0.5 mL/h).

To measure RBC aggregation in continuous blood flow, blood flowed sequentially through three channels with different channel widths such as the left wide channel, narrow channel, and right wide channel. Because RBC aggregation varied depending on shear rates, the shear rate for inducing or disaggregating RBC aggregation should be selected appropriately from the experimental results. Here, to stimulate RBC aggregation, the hematocrit of blood was adjusted to Hct = 50% by adding normal RBCs into a specific concentration of dextran solution of C_dextran_ = 10 mg/mL or PBS solution. A syringe pump was employed to supply the blood sample into a microfluidic channel, at a constant blood flow rate. As shown in Figure 2A, normal RBCs suspended in dextran solution increased image intensity (<I>) with decreasing shear rate ranging from $\dot{\gamma }=66.7{\;s}^{-1}$ to $\dot{\gamma }=12.5{\;s}^{-1}$. Insets show microscopic images captured with respect to shear rate ($\dot{\gamma }$) ((a) $\dot{\gamma }=12.5{\;s}^{-1}$, (b) $\dot{\gamma }=16.7{\;s}^{-1}$, and (c) $\dot{\gamma }=66.7{\;s}^{-1}$). The results indicated that RBC aggregation occurred at the specific shear rate of $\dot{\gamma }<66.7{\;s}^{-1}$. However, normal RBCs suspended in PBS solution did not show an increase in <I> with decreasing shear rates. Taking into account the fact that the syringe pump could provide constant blood flow rate consistently above 0.1 mL/h, the minimum value of blood flow rate was set to 0.1 mL/h. To determine channel widths of the wide channel and the narrow channel, blood flow rate was selected from Q_Blood_ = 0.1 mL/h to Q_Blood_ = 0.5 mL/h. Under constant values of blood flow rates, to induce RBC aggregation in the left wide channel and disaggregate RBC aggregation in the narrow channel, the widths of the wide channel (W_A_) and narrow channel (W_D_) were determined as W_A_ = 2000 μm and W_D_ = 100 μm, respectively. As shown in Figure 2B, the shear rates for the wide channel (W_A_) were estimated below $66.7{\;s}^{-1}$. The shear rates for the narrow channel (W_D_) were estimated above $66.7{\;s}^{-1}$. Thus, when blood flows under the constant value of flow rate ranging from 0.1 mL/h to 0.5 mL/h, RBCs tends to aggregate in the wide channel and disaggregate in the narrow channel distinctively.

### 3.2. Quantitative Evaluation of RBC Aggregation Index and Blood Viscosity under Constant Blood Flows

**Figure 3:**
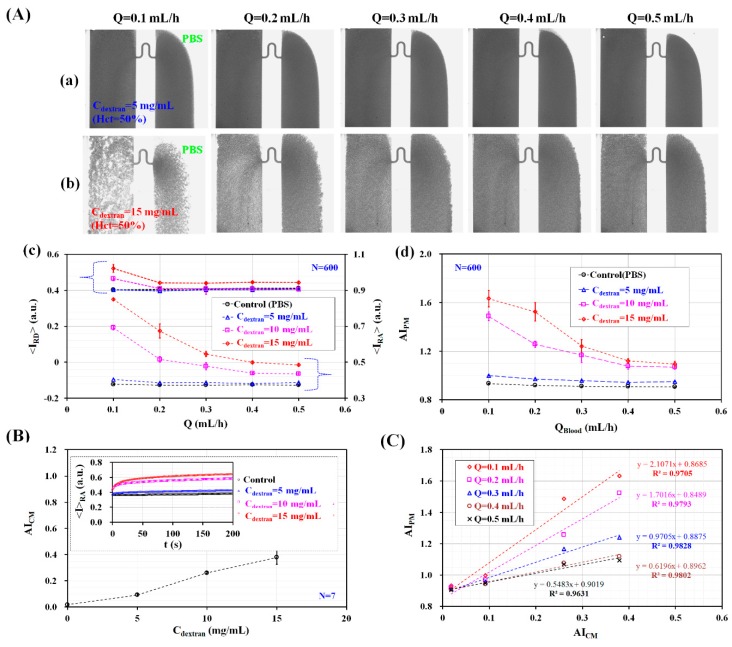
Quantitative evaluations of RBC aggregation index (AI_PM_) under constant blood flow. Blood (Hct = 50%) was prepared by adding normal RBCs into various concentrations of dextran solution (C_dextran_) (C_dextran_ = 0 mg/mL, 5 mg/mL, 10 mg/mL, and 15 mg/mL). Blood and PBS solution were delivered into the microfluidic device at the same flow rate (Q_PBS_ = Q_Blood_ = Q) (Q = 0.1 mL/h, 0.2 mL/h, 0.3 mL/h, 0.4 mL/h, and 0.5 mL/h). (**A**) Variations of RBC aggregation with respect to C_dextran_ and Q. (a) Microscopic images for representing RBC aggregation of blood (RBCs suspended in dextran solution of C_dextran_ = 5 mg/mL) with respect to Q. (b) Microscopic images for representing RBC aggregation of blood (RBCs suspended in dextran solution (C_dextran_ = 15 mg/mL)) with respect to Q. (c) Variations of <I_RA_> and <I_RD_> with respect to C_dextran_ and Q. (d) Variations of AI_PM_ with respect to C_dextran_ and Q. (**B**) Variations of conventional RBC aggregation index (AI_CM_) with respect to C_dextran_. Inset shows temporal variations of image intensity of RBCs with respect to C_dextran_, especially at stasis. (**C**) Quantitative comparison between AI_PM_ and AI_CM_. Regression coefficient of linear regression yielded higher value of R^2^ > 0.96 with respect to Q.

The RBC aggregation index (AI_PM_) was quantified by analyzing blood flows in microfluidic channels under constant blood flow. To stimulate the RBC aggregation of blood (Hct = 50%), normal RBCs were added into dextran solutions with varying concentration (C_dextran_ = 0 mg/mL, 5 mg/mL, 10 mg/mL, and 15 mg/mL). Here, C_dextran_ = 0 denotes a PBS solution. As shown in Figure 1A, blood and the PBS solution were simultaneously supplied into the microfluidic device at the same flow rate (Q_PBS_ = Q_Blood_ = Q, Q = 0.1 mL/h, 0.2 mL/h, 0.3 mL/h, 0.4 mL/h, and 0.5 mL/h) using two syringe pumps. As shown in Figure S1 (Supplementary Materials), microscopic images were captured for analyzing RBC aggregation for different concentrations of the dextran solution and blood flow rates. Among them, Figure 3A-a,b depicted microscopic images of blood prepared by adding normal RBCs into dextran solutions with two different concentrations (C_dextran_ = 5 mg/mL and 15 mg/mL) with respect to blood flow rate (Q).

At higher dextran solution concentrations, image intensity increased significantly, owing to RBC aggregation. However, it decreased when increasing the flow rate (or shear rate). By conducting digital image processing for microscopic images, the variations of <I_RA_> and <I_RD_> were obtained by varying C_dextran_ and Q. As shown in Figure 3A-c, for concentrations up to C_dextran_ = 5 mg/mL, <I_RA_> increased slightly by increasing C_dextran_ but remained constant with respect to Q. Above C_dextran_ = 5 mg/mL, <I_RA_> increased significantly with respect to C_dextran_ and decreased gradually at higher flow rates. When compared with <I_RA_>, <I_RD_> showed a similar behavior with respect to Q and C_dextran_. Quantitatively, the variation of <I_RD_> was much smaller than that of <I_RA_>. As shown in Figure 3A-d, temporal variations of AI_PM_ were obtained by dividing <I_RA_> by <I_RD_>. AI_PM_ increased slightly for concentrations up to C_dextran_ = 5 mg/mL. However, it remained constant with respect to Q. Above C_dextran_ = 5 mg/mL, AI_PM_ increased significantly with respect to the concentration of the dextran solution. However, it decreased gradually at higher flow rates. From these results, the RBC aggregation index (AI_PM_) varied significantly depending on the flow rate (Q) and the concentration of the dextran solution (C_dextran_). 

**Figure 4:**
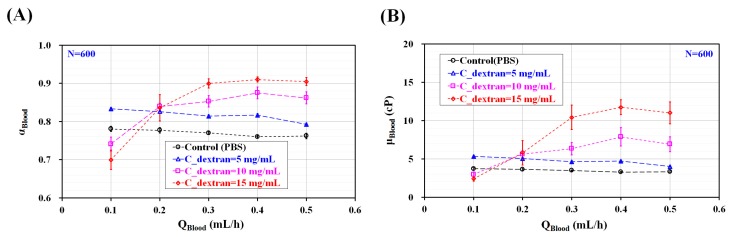
Quantitative evaluations of blood viscosity (µ_Blood_) under constant blood flow. Using various microscopic images as shown in Figure S1, α_Blood_ was obtained by varying blood flow rate (Q_Blood_) (Q_Blood_ = 0.1 mL/h, 0.2 mL/h, 0.3 mL/h, 0.4 mL/h, and 0.5 mL/h) and concentration of dextran solution (C_dextran_) (C_dextran_ = 0 mg/mL, 5 mg/mL, 10 mg/mL, and 15 mg/mL). (**A**) Variations of α_Blood_ with respect to Q_Blood_ and C_dextran_. (**B**) Variations of µ_Blood_ with respect to Q_Blood_ and C_dextran_.

To compare with the RBC aggregation index (AI_PM_) obtained by using the proposed method, the conventional aggregation index (AI_CM_) was quantified using the same blood. The previous method evaluated RBC aggregation by stopping blood flows. To measure RBC aggregation with the previous method [9], blood was supplied at a constant blood flow rate of Q = 1 mL/h. Blood flow was stopped immediately by clamping the tube connected to the driving syringe. Microscopic images were sequentially captured for specific duration of t_s_ = 200 s. Based on a specific ROI within the left wide channel, temporal variations of image intensity (<I_RA_>) were obtained by analyzing microscopic images. As shown in the inset of Figure 3B, the temporal variations of <I_RA_> increased gradually over time. In addition, it increased for higher concentrations of the dextran solution. The conventional RBC aggregation index (AI_CM_, AI_CM_ = S_A_/S_C_) [18] was obtained by analyzing the temporal variations of <I_RA_>. Here, S_A_ and S_C_ are denoted as $S_{A}=\int_{0}^{t_{s}}(\langle I_{\mathrm{RA}}\rangle -\langle I_{\mathrm{RA}}\left(t=0\right)\rangle )\mathrm{dt}$ and $S_{C}=\int_{0}^{t_{s}}\langle I_{\mathrm{RA}}\left(t=0\right)\rangle \mathrm{dt}$, respectively. When compared with the proposed method, the previous method was able to measure RBC aggregation in stationary blood flow. The variations of AI_CM_ were obtained by varying the concentration of the dextran solution, as shown in Figure 3B. AI_CM_ increased significantly when increasing the concentration of the dextran solution. The present method and previous method were compared by calculating two RBC aggregation indices (AI_PM_, and AI_CM_) for the same specific bloods consisting of different concentrations of dextran solution. As shown in Figure 3C, to evaluate the linear relationship between AI_PM_ and AI_CM_ with respect to C_dextran_, the corresponding indices for the values of Q used were spread in a scatter plot (AI_PM_ vs. AI_CM_). Because AI_PM_ was obtained in continuous blood flow, the linear relationship between AI_PM_ and AI_CM_ was quantified with respect to Q. According to our linear regression analysis, the coefficient of linear regression had a sufficiently high value of R^2^ > 0.97. These results indicated that AI_PM_ yielded consistent results when compared with AI_CM_. Thus, the RBC aggregation index (AI_PM_) proposed in this study can be effectively employed to monitor the variations of RBC aggregation, even in continuous blood flow. To quantify blood viscosity (µ_Blood_) for varying blood flow rates (Q_Blood_) and different concentrations of the dextran solution (C_dextran_), the variations in the blood-filled width (α_Blood_) were obtained by evaluating the interfacial location within a specific ROI, as depicted in Figure 1B. As shown in Figure S1 (Supplementary Materials). The interfacial location between the blood and the PBS solution was clearly detectable for concentrations up to C_dextran_ = 5 mg/mL and without respect to flow rate. However, with respect to C_dextran_ = 10 mg/mL and 15 mg/mL, the interface was not clearly detectable owing to excessive RBC aggregation. The excessive RBC aggregation made it difficult to detect the interfacial location consistently. When flow rate was set to above Q = 0.4 mL/h, the interfacial location was clearly detectable. As represented in Figure 4A, for concentrations up to C_dextran_ = 5 mg/mL, α_Blood_ increased significantly for higher concentrations of the dextran solution and decreased gradually when increasing the blood flow rate. For C_dextran_ = 10 mg/mL and 15 mg/mL, α_Blood_ increased significantly for flow rates up to Q = 0.4 mL/h. When the blood flow rate was increased from Q = 0.1 mL/h to Q = 0.4 mL/h, the RBC aggregates tended to break. Thus, the interfacial location was clearly detectable at higher blood flow rates. As shown in Figure 4B and based on Equation (2) and Figure 4A, the variations of blood viscosity were obtained with respect to Q and C_dextran_. For the left wide channel with extremely low aspect ratio (AR) (AR = h/w = 100/2000 = 0.05), the analytical formula of the shear rate is approximately derived as $\dot{\gamma }=\frac{6Q_{\mathrm{Blood}}}{{\mathrm{Wh}}^{2}}$. With respect to blood flow rate (Q_Blood_) (Q_Blood_ = 0.1 mL/h, 0.2 mL/h, 0.3 mL/h, 0.4 mL/h, and 0.5 mL/h), the corresponding shear rate of the blood flow rate was estimated as $\dot{\gamma }=8.3{\;s}^{-1\;}\left(Q_{\mathrm{Blood}}=0.1\frac{\mathrm{mL}}{h}\right)$, $16.7{\;s}^{-1\;}\left(Q_{\mathrm{Blood}}=0.2\frac{\mathrm{mL}}{h}\right)$, $\;25.0{\;s}^{-1\;}\left(Q_{\mathrm{Blood}}=0.3\frac{\mathrm{mL}}{h}\right)$, $\;33.3{\;s}^{-1\;}\left(Q_{\mathrm{Blood}}=0.4\frac{\mathrm{mL}}{h}\right)$, and $41.7{\;s}^{-1\;}\left(Q_{\mathrm{Blood}}=0.5\frac{\mathrm{mL}}{h}\right)$. For C_dextran_ = 0 mg/mL and 5 mg/mL, blood viscosity decreased gradually when increasing the blood flow rate. However, for C_dextran_ = 10 mg/mL and 15 mg/mL, the blood viscosity was measured consistently for flow rates above Q = 0.4 mL/h. When the blood flow rate was set to be above 0.4 mL/h, blood viscosity increased significantly by increasing the concentration of the dextran solution. From these results, the modified parallel flow method (MPFM) did not provide consistent values of blood viscosity when the excessive RBC aggregation occurred. To quantify blood viscosity in such cases with excessive RBC aggregation, the blood flow rate should be increased to detect the interfacial location between the two fluids clearly.

### 3.3. Quantitative Evaluation of RBC Aggregation Index and Blood Viscosity under Pulsatile Blood Flow

The RBC aggregation index and blood viscosity were simultaneously obtained for pulsatile blood flow. Blood was prepared by adding normal RBCs into a dextran solution with a specific concentration (C_dextran_ = 10 mg/mL). As shown in Figure 3A-d and based on our experimental results on the RBC aggregation index (AI_PM_) with respect to blood flow rate, the maximum blood flow rate (Q_max_) and minimum blood flow rate (Q_min_) were set to Q_max_ = 0.5 mL/h and Q_min_ = 0.2 mL/h, respectively. RBCs aggregated at lower flow rates, but these aggregates were broken at higher blood flow rates. By controlling the two syringe pumps, the blood and the PBS solution were periodically supplied into the microfluidic device at the same flow rate (Q_PBS_ = Q_Blood_ = Q). Here, the flow rate (Q) was set to 0.5 mL/h from t = 0 to t < 0.5T. Additionally, Q was set to 0.2 mL/h from t = 0.5T to t = T. Thus, during these pulsatile blood flows, RBCs aggregated and disaggregated depending on the magnitude of the blood flow rate.

**Figure 5:**
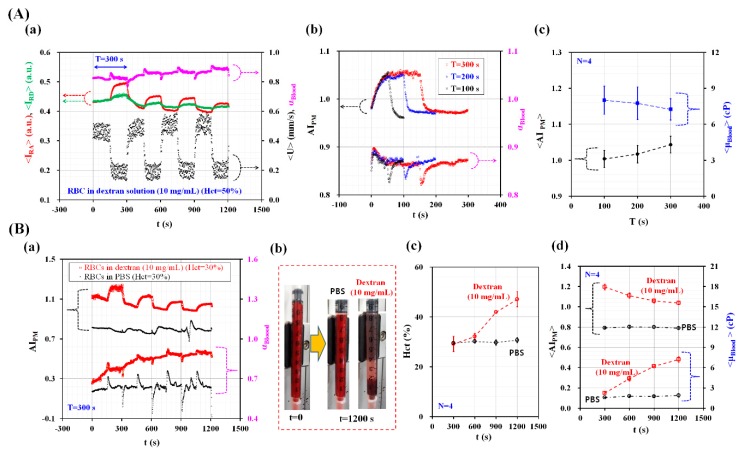
Quantitative evaluations of RBC aggregation index and blood viscosity under pulsatile blood flow conditions (Q_max_ = 0.5 mL/h, Q_min_ = 0.2 mL/h, and period (T)). Blood and PBS solution were periodically supplied into the microfluidic device, at the same flow rate. (**A**) Quantitative evaluation of the effect of period (T) on RBC aggregation index and blood viscosity for blood (Hct = 50%). Blood (Hct = 50%) was prepared by adding normal RBCs into specific concentration of dextran solution (C_dextran_ = 10 mg/mL). (a) Variations of <I_RA_>, <I_RD_>, α_Blood_, and <U> over time. (b) Temporal variations of AI_PM_ and α_Blood_ with respect to period (T) (T = 100 s, 200 s, and 300 s). (c) Variations of <AI_PM_> and <α_Blood_> with respect to T. Here, the <AI_PM_> was obtained by averaging variations of AI_PM_ for the corresponding duration of Q = 0.2 mL/h. The <α_Blood_> was obtained by averaging α_Blood_ for a single period. (**B**) Quantitative evaluation of RBC aggregation index and blood viscosity for blood (RBCs suspended into two base solutions (PBS solution and 10 mg/mL concentration of dextran solution)). The period was fixed at T = 300 s. Hematocrit of two bloods were adjusted to 30%. (a) Temporal variations of AI_PM_ and μ_Blood_ with respect to base solution. (b) RBC sedimentation in a syringe depending on two base solutions after 1200 s were elapsed. (c) Variations of Hct of blood collected at the outlet (a) depending on two base solutions, at intervals of 300 s. (d) Variations of <AI_PM_> and <μ_Blood_> depending on two base solutions, at intervals of 300 s.

**Figure 6:**
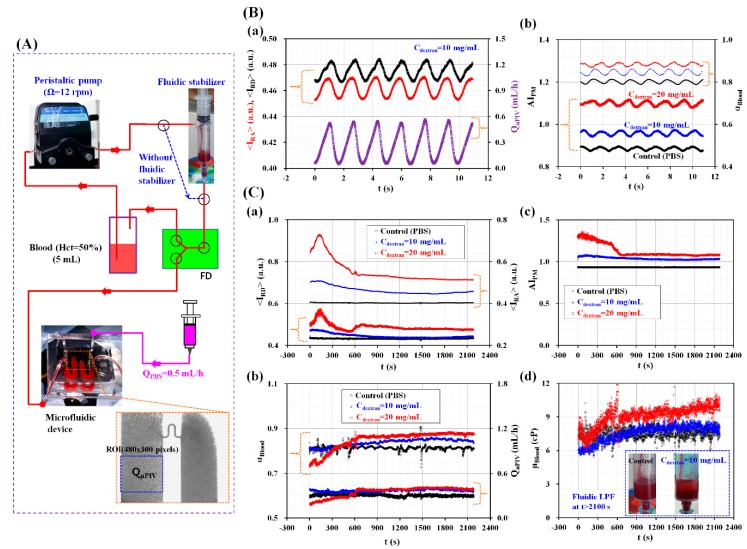
Continuous and simultaneous measurement of RBC aggregation and blood viscosity of blood circulating within an in vitro closed fluidic circuit. (**A**) Schematic diagram of the fluidic circuit constructed by connecting several components such as a peristaltic pump, a fluidic stabilizer, fluid divider (FD), a microfluidic device and reservoir, and a syringe pump. (**B**) Quantitative evaluation of RBC aggregation index and blood-filled width depending on various concentrations of dextran solution, under periodic blood flow conditions. (a) Temporal variations of <I_RA_>, <I_RD_>, and Q_μPIV_ for blood (RBCs suspended in dextran solution (C_dextran_ = 10 mg/mL)). (b) Temporal variations of AI_PM_ and α_Blood_ with respect to C_dextran_ (C_dextran_ = 0 mg/mL, 10 mg/mL, and 20 mg/mL). (**C**) Quantitative evaluation of RBC aggregation index (AI_PM_) and blood viscosity (µ_Blood_) with respect to C_dextran_, especially in constant blood flow. Since the fluidic stabilizer removed pulsatile blood flows, blood flow remained constant in the microfluidic device. (a) Temporal variations of <I_RD_> and <I_RA_> with respect to C_dextran_. (b) Temporal variations of AI_PM_ with respect to C_dextran_. (c) Temporal variations of α_Blood_ with respect to C_dextran_. (d) Temporal variations of μ_Blood_ with respect to C_dextran_. Inset shows RBC sedimentation in the fluidic stabilizer depending on base solutions (PBS solution, and dextran solution (C_dextran_ = 10 mg/mL)) after 2100 s were elapsed.

As shown in Figure 5A-a, the temporal variations of <I_RA_>, <I_RD_>, α_Blood_, and <U> were obtained over 1200 s. The period of the blood flow rate was set to T = 300 s. Blood (Hct = 50%) was prepared by adding normal RBCs into a dextran solution (C_dextran_ = 10 mg/mL). <U> denotes the averaged blood velocity calculated within a specific ROI (480 × 300 pixels) in the left wide channel, as depicted in Figure 6A. <I_RA_> increased for lower blood velocities. However, it decreased at higher blood velocities. In addition, <I_RD_> showed similar behavior when compared with <I_RA_>. α_Blood_ increased for the lower blood flow rate because of its shear-thinning behavior. As shown in Figure 5A-b, the values of AI_PM_ and α_Blood_ over multiple periods were overlapped (T = 100 s, 200 s, and 300 s) to inspect periodic behaviors. At T = 100 s, AI_PM_ and α_Blood_ did not reach steady values because of their long transient behavior. When increasing the period of the blood flow rate to T = 200 s and 300 s, they did reach a peak value and remained constant over time. To quantify the effects of period on AI_PM_ and µ_Blood_, the averaged RBC aggregation index (<AI_PM_>) was obtained by averaging the variations of AI_PM_ for the corresponding duration of Q = 0.2 mL/h ($\langle {\mathrm{AI}}_{\mathrm{PM}}\rangle =\frac{1}{0.5T}\int_{0.5T}^{T}{\mathrm{AI}}_{\mathrm{PM}}\mathrm{dt}\;$). Additionally, the averaged blood viscosity (<µ_Blood_>) was obtained by averaging the variations of µ_Blood_ over a single period ($\langle {\text{µ}}_{\mathrm{Blood}}\rangle =\frac{1}{T}\int_{0}^{T}{\text{µ}}_{\mathrm{Blood}}\;\mathrm{dt}\;$). As shown in Figure 5A-c, the variations of <AI_PM_> and <µ_Blood_> were obtained by varying the period (T = 100 s, 200 s, and 300 s). <AI_PM_> increased gradually when increasing the period. Additionally, <µ_Blood_> decreased with respect to the period. These results indicate that the RBC aggregation index and blood viscosity varied depending on the period of the blood flow rate. The effects of the base solution (the PBS solution and the dextran solution (C_dextran_ = 10 mg/mL)) on the RBC aggregation index and blood viscosity were quantified as shown in Figure 5B. The period was fixed at T = 300 s. The hematocrit of two blood batches was adjusted to Hct = 30% by adding normal RBCs into two base solutions. As shown in Figure 5B-a, the temporal variations of AI_PM_ and α_Blood_ were obtained over 1200 s. As time elapsed, AI_PM_ for the dextran solution significantly decreased over time compared with that for the PBS solution. However, this led to an increase in blood viscosity (α_Blood_) over time. As shown in Figure 5B-b, RBC sedimentation in one of the driving syringes was visualized after an elapse of 1200 s. The RBCs suspended in the dextran solution were separated from the base solution. They were stacked at a lower position (near the bottom) when compared with the RBCs suspended in the PBS solution. To monitor the variations of the hematocrit over time, they were obtained for both the PBS solution and the dextran solution. 

After collecting blood from outlet (a) (Figure 1A) for 300 s, the hematocrit of the collected blood was measured using a micro hematocrit (VS-12000, Vision Scientific, Daejeon, Korea). As shown in Figure 5B-c, the variations of the Hct were monitored at intervals of 300 s. The RBCs suspended in the PBS solution did not vary the hematocrit level over time. However, the RBCs suspended in the dextran solution increased the hematocrit level significantly owing to RBC aggregation in the driving syringe. As shown in Figure 5B-d, the temporal variations of <AI_PM_> and <μ_Blood_> were obtained at intervals of 300 s. As demonstrated by our results, the dextran solution increased RBC aggregation more over time when compared with the PBS solution. For this reason, the RBCs suspended in the dextran solution decreased the <AI_PM_> but increased <μ_Blood_> as time elapsed. These experimental results leads us to the conclusion that the proposed method has the ability to consistently quantify RBC aggregation and blood viscosity even under pulsatile blood flows.

### 3.4. Quantitative Evaluation of RBC Aggregation and Blood Viscosity under an In-Vitro Fluidic Circuit

In this last demonstration, the proposed method was adopted to measure the RBC aggregation and viscosity of blood circulating within an in vitro closed fluidic circuit, particularly in a continuous and simultaneous fashion. As shown in Figure 6A, the closed fluidic circuit was constructed by connecting several components such as a peristaltic pump, a fluidic stabilizer, a fluid divider (FD), a microfluidic device, a reservoir, and a syringe pump [9]. The air cavity was adjusted to 2.5 mL inside the fluidic stabilizer. Without the fluidic stabilizer, the blood flow varied in a peristaltic fashion because of the peristaltic pump used. When blood passed through the fluidic stabilizer, the air compliance inside the fluidic stabilizer removed the pulsatile components of the blood flow [9,44,45]. Blood was then supplied into the FD (width = 3000 µm and depth = 100 µm) at constant blood flow. From the outlet of the FD, blood was supplied into inlet (a) of the microfluidic device. Unlike a previous study [9], a pinch valve was not employed to control the blood flow (for realizing a periodic on-off control). The peristatic pump was set to Ω = 12 rpm. The PBS solution was supplied into inlet (b) of the microfluidic device at a flow rate of Q_PBS_ = 0.5 mL/h. The blood flow rate (Q_μPIV_) was arithmetically calculated by multiplying the averaged blood velocity (<U>) by the cross-sectional area (A_c_) (Q_μPIV_ = <U> × A_c_). To stimulate RBC aggregation, various blood batches were prepared by adding normal RBCs into dextran solutions with specific concentrations (C_dextran_ = 0 mg/mL, 10 mg/mL, and 20 mg/mL). The reservoir was filled with a blood volume of 5 mL.

As shown in Figure 6B, the temporal variations of the RBC aggregation index (AI_PM_) and the blood-filled width (α_Blood_) were obtained by varying the concentration of the dextran solution. As shown in Figure 6A, the outlet of the peristaltic pump was directly connected to the inlet of the FD via a polyethylene tube. To obtain the blood velocity fields, two consecutive microscopic images were captured at intervals of 50 ms. Figure 6B-a shows the temporal variations of <I_RA_>, <I_RD_>, and Q_μPIV_ for the blood batches (Hct = 50%) prepared by adding normal RBCs into a dextran solution with a concentration of C_dextran_ = 10 mg/mL. Here, the <I_RA_> and <I_RD_> showed periodic behaviors depending on the blood flow rate (Q_µPIV_). In addition, they exhibited negligible phase differences when compared with the blood flow rate. As shown in Figure 6B-b, the temporal variations of AI_PM_ and α_Blood_ were obtained by varying C_dextran_. AI_PM_ increased significantly when the concentration of the dextran solution was increased. However, when compared with the case of using a constant blood flow rate (Figure 3A), AI_PM_ decreased significantly for periodic blood flows. Periodic blood flows reduced RBC aggregation significantly. Additionally, α_Blood_ increased significantly when increasing C_dextran_. By referring to Equation (2), blood viscosity increased for dextran solutions with a higher concentration. From these experimental results, we concluded that the proposed method could effectively and simultaneously measure variations in RBC aggregation and blood viscosity under periodic blood flows.

On the other hand, by applying the fluidic stabilizer as shown in Figure 6C, the RBC aggregation index (AI_PM_) and blood viscosity (µ_Blood_) were simultaneously measured with respect to C_dextran_ (0 mg/mL, 10 mg/mL, and 20 mg/mL) under a constant blood flow rate. As shown in Figure 6C-a, the temporal variations of <I_RD_> and <I_RA_> were obtained by varying C_dextran_. <I_RD_> and <I_RA_> increased for higher concentrations of the dextran solution. In addition, they showed transient behaviors over 600 s. Thereafter, <I_RD_> and <I_RA_> remained constant without respect to time. As shown in Figure 6C-b, the temporal variations of α_Blood_ and Q_µPIV_ were simultaneously obtained by increasing C_dextran_. After 600 s had elapsed, both of them remained constant with respect to time. Using the obtained temporal variations of <I_RD_> and <I_RA_> and as shown in Figure 6C-c, the continuous temporal variations of AI_PM_ were obtained with respect to C_dextran_. For blood batches with C_dextran_ = 10 mg/mL and 20 mg/mL, AI_PM_ decreased significantly over up to 600 s. Then, it remained constant over time. However, for blood batches mixed with the PBS solution, AI_PM_ remained constant over time. As shown in Figure 6C-d, the temporal variations of blood viscosity (µ_Blood_) were continuously obtained by increasing C_dextran_. The inset of Figure 6C-d shows RBC sedimentation in the fluidic stabilizer, which occurred depending on the base solution used (either the PBS solution or the dextran solution (C_dextran_ = 10 mg/mL)) after 2100 s had elapsed. The control blood (RBCs suspended in the PBS solution) did not distinctively show RBC sedimentation. However, for blood made using a dextran solution with a concentration of 10 mg/mL, the RBCs were clearly separated from the base solution owing to RBC sedimentation. As a result, the blood made using the PBS solution showed a constant blood viscosity after 600 s had elapsed. However, for blood batches made using dextran solutions, blood viscosity increased gradually over time because RBC sedimentation caused increases in the hematocrit levels.

From these experimental demonstrations, the proposed method was successfully used to measure RBC aggregation and blood viscosity without interrupting the circulation of blood in the closed fluidic circuit and, more importantly, in a continuous and simultaneous fashion.

## 4. Conclusions

In this study, a simple method for measuring RBC aggregation and blood viscosity was proposed, which consists of analyzing blood flows in a microfluidic device in a continuous and simultaneous fashion. To demonstrate this method, a microfluidic device was designed to have a left wide channel, a narrow channel, a right wide channel, inlets, and outlets. To measure RBC aggregation, blood sequentially flowed through the three channels with different widths such as the left wide channel, the narrow channel, and finally the right wide channel. When compared with the image intensity of the RBCs aggregated in the left channel (<I_RA_>), the image intensity of the RBCs disaggregated within the specific ROI of the right channel (<I_RD_>) was lower owing to RBC disaggregation. Then, the RBC aggregation index (AI_PM_) was defined as a new index, which is obtained by dividing <I_RA_> by <I_RD_> (AI_PM_ = <I_RA_>/<I_RD_>). Simultaneously, by supplying the PBS solution into the right wide channel, blood viscosity was measured by detecting the interfacial location between the two fluids. First, RBC aggregation and blood viscosity were evaluated under constant blood flow rates. The RBC aggregation index (AI_PM_) varied significantly depending on the flow rate used (Q = 0.1 mL/h, 0.2 mL/h, 0.3 mL/h, 0.4 mL/h, and 0.5 mL/h) and the concentration of the dextran solution (C_dextran_ = 0 mg/mL, 5 mg/mL, 10 mg/mL, and 15 mg/mL). The RBC aggregation indexes obtained using the proposed method were quantitatively compared with those obtained via the previous method (AI_CM_). According to our linear regression analysis, the coefficient of linear regression yielded a sufficiently high value of R^2^ > 0.97. These results indicate that the AI_PM_ provided consistent results when compared with AI_CM_. Blood viscosity was simultaneously obtained by evaluating the blood-filled width (α_Blood_) in the right wide channel. The modified parallel flow method (MPFM) did not provide consistent values of blood viscosity when the blood underwent excessive RBC aggregation. After the blood flow rate was increased to detect the interface clearly, blood viscosity could be obtained consistently even after excessive RBC aggregation. Secondly, under periodic pulsatile blood flows (maximum flow rate = 0.5 mL/h, minimum flow rate = 0.2 mL/h), the variations of RBC aggregation index and blood viscosity were evaluated by varying the period (T = 100 s, 200 s, and 300 s) and the base solution as the dextran solution. The RBC aggregation index and blood viscosity varied depending on the period of the pulsatile blood flows. Using the dextran solution increased RBC sedimentation, which resulted in an increase in the hematocrit levels over time. Thus, the RBCs suspended in the dextran solution had <AI_PM_> values that decreased but <μ_Blood_> values that increased over time. In the last demonstration, the suggested method was employed to obtain the RBC aggregation index and blood viscosity of blood batches circulating within an in vitro fluidic circuit. From our results, the proposed method could successfully measure RBC aggregation and blood viscosity in a continuous and simultaneous fashion without interrupting the blood flow (without on-off blood control). 

## Figures and Tables

**Table 1:** Summary of microfluidic-based technique for measuring red blood cell (RBC) aggregation and blood viscosity (O and X denote possible method and impossible method, respectively).

RBC Aggregation		Blood Viscosity		Simultaneous	Comments	Ref.
Methods	Continuous	Methods	Continuous			
Photometric intensity	X			X	-Vacuum pump -Light backscattering or transmission	[19,20]
	X			X	-Pinch valve -Light transmission	[15]
Electric impedance	X			X	-Pipette [21] or syringe pump [22] -Conductivity	[21,22]
Ultrasonic imaging	X			X	-Syringe pump -Speckle size	[23]
Microscopic imaging	X			X	-Pressure system -Aggregate size	[26]
	X			X	-Syringe pump -Cluster size or occurrence	[25]
	X			X	-Inverted syringe pump -intensity variations	[27]
	Periodic (T = 4–5 min)			X	-Air-suction pump [18] or syringe pump [16,17] (on-off blood flows) -Intensity variations	[16,17,18]
	Periodic (T = 4 min)	Modified parallel flows	O	O	-Two syringe pumps (on-off blood flows) -Intensity variations -Interfacial locations	[24]
	Periodic (T = 400 s)	Modified parallel flows	O	O	-Two syringe pumps (on-off blood flows) -Intensity variations -Interfacial locations	[9]
		Co-flowing streams	Periodic	X	-Two syringe pumps -Flow rate for relocating interface to the center	[34]
		Microflow compartment	O	X	-Two syringe pumps -Numbers of channels filled with each fluid at the same flow rate	[35,36]
		Reversal flow switching	Periodic	X	-Two syringe pumps -Flow rate for inducing reversal flow in the bridge channel	[10,11,37,38,39]
		Advancing meniscus	X	X	-Surface tension-driven blood flow -Mean velocity	[29,30,31,32]
			X	X	-Blood delivery (pressure [28] or pipette [33]) -Smartphone-based imaging	[28,33]
		Electric impedance	O	X	-Syringe pump -Electric resistance	[40,41]

## Supplementary Materials

The following are available online at http://www.mdpi.com/2072-666X/9/9/467/s1, Figure S1: Microscopic images of blood flow with respect to blood flow-rate (Q_Blood_) and concentrations of dextran solution (C_dextran_).
