# Supplementary material for: Microfluidic-Based Technique for Measuring RBC Aggregation and Blood Viscosity in a Continuous and Simultaneous Fashion
Source: Micromachines (Basel). 2018 Sep 14;9(9):467. doi: 10.3390/mi9090467 (PMC6187833; doi:10.3390/mi9090467)
Supplement: Supplementary file 1 [file micromachines-09-00467-s001.pdf]

Supplementary Materials: Microfluidic-based method for measuring RBC aggregation and blood viscosity in a continuous and simultaneous fashion

Yang Jun Kang

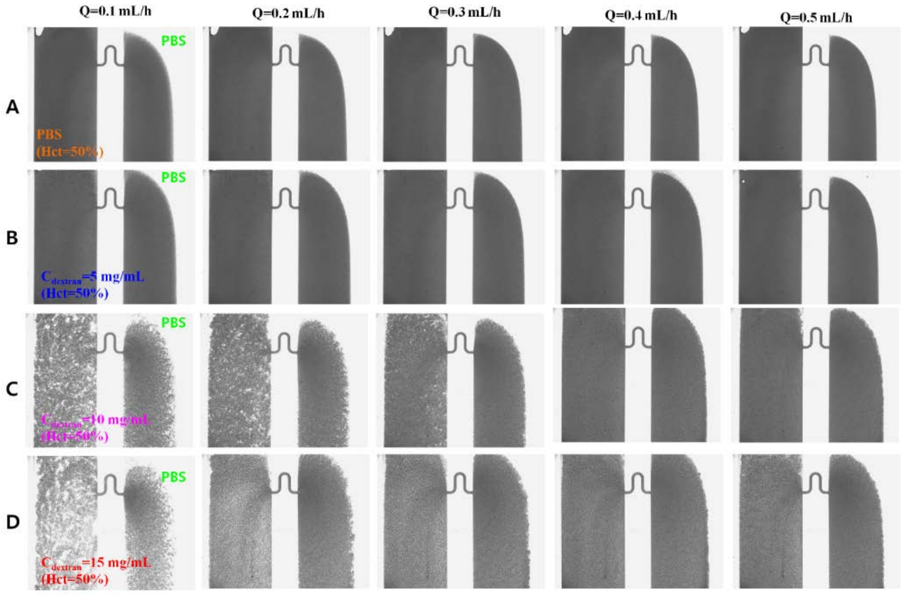

Figure S1. Microscopic images of blood flow with respect to blood flow rate (Q_{Blood}) and concentrations of dextran solution ($C_{dextran}$). Hematocrit of blood was adjusted to 50% by adding normal RBCs into a specific concentration of dextran solution ($C_{dextran}$) ($C_{dextran}$ = 0 mg/mL, 5 mg/mL, 10 mg/mL, and 15 mg/mL). Blood and PBS solution were simultaneously supplied into the microfluidic device, at the sample flow rate (Q_{Blood}=Q_{PBS}=Q). **(A)** Microscopic images representing RBC aggregation of blood (RBCs suspended in PBS solution) with respect to Q. **(B)** Microscopic images representing RBC aggregation of blood (RBCs suspended in dextran solution ($C_{dextran}$= 5 mg/mL)) with respect to Q. **(C)** Microscopic images representing RBC aggregation of blood (RBCs suspended in dextran solution ($C_{dextran}$= 10 mg/mL)) with respect to Q. **(D)** Microscopic images representing RBC aggregation of blood (RBCs suspended in dextran solution ($C_{dextran}$= 15 mg/mL)) with respect to Q.
